# Metabolic Analysis of Nucleosides/Bases in the Urine and Serum of Patients with Alcohol-Associated Liver Disease

**DOI:** 10.3390/metabo12121187

**Published:** 2022-11-28

**Authors:** Liqing He, Vatsalya Vatsalya, Xipeng Ma, Carolyn M. Klinge, Matthew C. Cave, Wenke Feng, Craig J. McClain, Xiang Zhang

**Affiliations:** 1 Department of Chemistry, University of Louisville, Louisville, KY 40292, USA; 2 Alcohol Research Center, University of Louisville School of Medicine, Louisville, KY 40202, USA; 3 Hepatobiology & Toxicology Center, University of Louisville School of Medicine, Louisville, KY 40202, USA; 4 Center for Regulatory and Environmental Analytical Metabolomics, University of Louisville, Louisville, KY 40292, USA; 5 Department of Medicine, University of Louisville School of Medicine, Louisville, KY 40202, USA; 6 Department of Biochemistry and Molecular Genetics, University of Louisville School of Medicine, Louisville, KY 40202, USA; 7 Department of Pharmacology & Toxicology, University of Louisville School of Medicine, Louisville, KY 40202, USA; 8 Robley Rex Department of Veterans Affairs Medical Center, Louisville, KY 40206, USA

**Keywords:** modified nucleosides, 7,9-dimethylguanine, ms^2^t^6^A, epitranscriptomics, alcohol-associated liver disease

## Abstract

Accumulating evidence supports the important role of RNA modifications in liver disease pathogenesis. However, RNA modifications in alcohol-associated liver disease (ALD) have not yet been reported. Modified ribonucleosides/bases are products of RNA degradation; therefore, we investigated whether modified ribonucleosides/bases in human urine and serum are changed and whether these changes are associated with the severity of ALD. Human urine and serum samples from patients with ALD and appropriate controls were collected. Free nucleosides/bases were extracted from these samples and quantified using untargeted and targeted metabolomic approaches. Thirty-nine and forty free nucleosides/bases were respectively detected in human urine and serum samples. Twelve and eleven modified nucleosides are significantly changed in patients’ urine and serum (*q* < 0.05 and fold-change > 20%). The abundance of modified nucleobase and ribonucleoside, 7,9-dimethylguanine in urine and 2-methylthio-*N*^6^-threonylcarbamoyladenosine (ms^2^t^6^A) in serum are strongly associated with the severity of ALD. Spearman’s rank correlation coefficient of these two metabolites with the Model for End-stage Liver Disease (MELD) score are 0.66 and 0.74, respectively. Notably, the abundance changes in these two metabolites are sufficiently large to distinguish severe alcohol-associate hepatitis (AH) from non-severe ALD and non-severe ALD from healthy controls.

## 1. Introduction

Various chemical modifications have been identified in RNA molecules of living organisms, and 163 modifications have been recorded in the MODOMICS database [1]. Chemical modifications in RNA molecules have diverse functions; they affect the structure, stability, and maturation of RNA and then diversify their functions in gene expression. For instance, *N*^6^-methyladenosine (m^6^A), the most extensively studied RNA modification, regulates protein translation rate [2], RNA alternative splicing, and RNA metabolism [3]. m^6^A modification participates in multiple biological processes, including embryonic [4] and cerebellum development [5], and hematopoietic and progenitor cell specification [6]. RNA modification also plays a significant role in the pathogenesis of human diseases. m^6^A demethylation on forkhead box M1 (FOXM1) mRNA maintains the tumorigenesis of glioblastoma stem-like cells [7]. Fat-mass and obesity-associated protein (FTO), an m^6^A demethylase, enhances leukemic oncogene-mediated cell transformation and leukemogenesis [8]. Other RNA modifications, such as 2-thiouridine (s^2^U) and 2′-O-methylguanosine (Gm), also play an important role in the pathogenesis of human diseases [9].

The liver is a crucial metabolic and digestive organ and plays a key role in many pathophysiological processes. m^6^A RNA modification in the liver is highly related to hepatic function and the development of liver diseases [10]. m^6^A regulates adipogenesis in non-alcoholic fatty liver disease [11,12], virus replication, and particle production in hepatitis B and C [13,14]. The imbalance of A to inosine (I) RNA editing increases the risk of liver cirrhosis [15]. The changes in mitochondrial tRNA modification cause acute infantile liver failure [16,17]. *N*^2^,*N*^2^-dimethylguanosine (m^2,2^G) and *N*^1^-methylinosine (m^1^I) are higher in the serum and urine of patients who developed acute kidney injury (AKI) from advanced cirrhosis and required dialysis [18]. Another modified ribonucleoside, 1-methyladenosine (m^1^A), is closely related to the development of hepatocellular carcinoma (HCC). The mutation of m^1^A-related regulatory genes, m^1^A writers, readers, and erasers, may play crucial roles in regulating HCC progression, and increased expression of m^1^A writers and readers is found in HCC patients [19]. We identified ten significant RNA modifications that were regulated by polychlorinated biphenyl and high-fat diet exposures in total liver RNA in a mouse model of non-alcoholic fatty liver disease [20]. Increases in *N*^6^, 2’-O-dimethyladenosine (m^6^Am) and pseudouridine (Ψ) are associated with increased PCIF1 and PUS10 writer protein expression, while increased m^1^A and m^6^A are associated with increased reader YTHDF2 and YTHDC2 transcript and reader FMR1 transcript and protein abundance [20]. However, there has not yet been reported whether RNA modification changed in alcohol-associated liver disease (ALD) and whether these changes could serve as markers for ALD diagnosis.

RNA is synthesized during gene transcription, and its chemical modification happens during maturation. At the end of its life, the modified RNA is degraded into single ribonucleosides, resulting in modified ribonucleosides excreted to extracellular spaces, circulated in serum, and discarded into the urine. This study aimed to investigate whether RNA chemical modification is changed in ALD and whether the modified ribonucleosides in urine and serum can serve as markers for the early-stage ALD diagnosis. We collected urine and serum samples from ALD patients and healthy volunteers. Free nucleosides/bases were extracted from those samples and analyzed by untargeted and targeted metabolomics using two analytical platforms, i.e., parallel two-dimensional liquid chromatography-mass spectrometry (2DLC-MS), and solid phase extraction followed by reversed-phase chromatography-mass spectrometry (SPE RPC-MS) [21,22].

## 2. Methods and Samples

### 2.1. Chemicals and Reagents

Seventy-eight authentic standards of ribonucleosides/bases were purchased from Toronto Research Chemicals Inc. (Toronto, ON, Canada), Cayman Chemical Company (Ann Arbor, MI, USA), and Biosynth International, Inc. (San Diego, CA, USA).

### 2.2. Patient Recruitment and Sample Collection

The clinical study was approved by the University of Louisville Institutional Review Board. The information on patient definitions and recruitment is detailed in another publication [23]. All study participants provided informed consent before participation, including appropriate data and sample collection authorization. Nineteen healthy controls (HC) and 46 ALD patients were enrolled in this observational single-timepoint study. Patients with non-severe ALD (*n* = 21) were comprised of patients with moderate alcohol-associated hepatitis (AH, 12 ≤ MELD < 20) and patients with alcohol use disorder and abnormal liver enzymes (MELD < 12). Severe AH patients (*n* = 25) were defined as those with MELD ≥ 20. All study participants had a complete history, physical examination, and laboratory evaluation upon enrollment. Patient samples were categorized by MELD score, and the group information is listed in Table 1.

All participants’ specimens (urine and blood) were collected in the morning after overnight fasting and stored at −80 °C until use. All de-identified data from participants who provided specimens were collected at baseline, and information on subsequent death was provided, if available. Clinical data included participant demographics (age, sex), drinking history, medical assessments at admission, and medical history. Confirmatory tests for AH (laboratory and imaging) and assessment of liver severity (MELD score) were collected and analyzed. A laboratory panel specific to this study was comprised of a comprehensive metabolic panel (CMP, including liver injury panel), coagulation measures, and complete blood count tests. All data were analyzed at the University of Louisville.

### 2.3. Metabolite Extraction

Free nucleosides/bases and other polar metabolites were extracted using the method described previously [21,22]. One hundred µL of human urine and 50 µL of serum sample were used for metabolite extraction.

### 2.4. LC-MS/MS Analysis

All samples were analyzed in random order on a Thermo Q Exactive HF Hybrid Quadrupole-Orbitrap Mass Spectrometer coupled with a Thermo DIONEX UltiMate 3000 HPLC system (Thermo Fisher Scientific, Waltham, MA, USA). For untargeted metabolic profiling, the UltiMate 3000 HPLC system was equipped with a parallel 2DLC (a reversed-phase chromatography (RPC) column and a hydrophilic interaction liquid chromatography (HILIC) column) [22]. For the targeted metabolomic study, the UltiMate 3000 HPLC system was equipped with an RPC column [21]. To obtain full MS data, every sample was analyzed by LC-MS in positive and/or negative modes depending on the chemical properties of the metabolites of interest. One pooled sample of each group was analyzed by LC-MS/MS to acquire MS/MS spectra at three collision energies (20, 40, and 60 eV) for metabolite identification.

### 2.5. Data Analysis

XCMS software was used for spectrum deconvolution [24]. For metabolite identification, the m/z, retention time, and MS/MS spectra of the 78 authentic standards of ribonucleosides were uploaded into mzVault, a component of Compound Discovery software (version 3.2, Thermo Fisher Scientific, Inc., Dreieich, Germany), as an in-house database. Compound Discovery software was used for metabolite identification by matching the experimental data to the information of the 78 authentic standards (i.e., parent ion *m*/*z*, retention time, and tandem MS spectrum) and the MS/MS spectra of other nucleosides in the Compound Discovery software. The threshold of spectrum matching was set to *m*/*z* variation ≤ 5 ppm, retention time variation ≤ 0.15 min, and spectrum similarity score ≥ 0.5.

Statistical analyses were performed using SPSS software (version 25, IBM Corporation, Armonk, NY, USA). Distributional assumptions of continuous outcomes were checked, and, if needed, a data transformation (e.g., log-transformation) was applied to meet the normality assumption. Univariate analysis of metabolite abundance among groups was conducted using one-way ANOVA with Tukey post-hoc test, and the Benjamini and Hochberg method [25] was used for multiple testing correction. Receiver operating characteristic (ROC) analysis was used to illustrate the diagnostic ability of ALD patients as the metabolite abundance varies. Linear-by-linear association test was used to study the association between the abundance of a metabolite and the severity of ALD. Spearman’s rank correlation was used to measure the association of each metabolite with the clinical parameter MELD score. The thresholds for all tests were set as follows: one-way ANOVA *q* ≤ 0.05, areas under the ROC curve (AUC) > 0.7 or <0.3 and *p* ≤ 0.05, linear-by-linear association test *p* ≤ 0.05, and Spearman’s rank correlation coefficient |*ρ*| ≥ 0.4. The error bars in each histogram plot are the standard error of the mean (SEM).

## 3. Results

### 3.1. Modified Nucleosides/Bases Are Increased in the Urine of Patients with ALD

The information of all 65 participants is provided in Table 1. We categorized all samples into three groups: healthy controls (HC, *n* = 19), non-severe ALD (MELD < 20, *n* = 21), and severe AH (MELD ≥ 20, *n* = 25). We first analyzed the abundance changes of polar metabolites (including free nucleosides/bases) in human urine samples using 2DLC-MS via untargeted metabolomics [22]. Eight thousand features (i.e., isotopic peaks) were detected. Four hundred seventy-nine metabolites were identified by matching experimental data to our in-house database and the public database. Among the 479 identified metabolites, 25 were nucleosides/bases, and 16 were identified from our in-house database.

One-way ANOVA shows that 10 nucleosides/bases are significantly changed with *q* < 0.05 and fold-change (FC) > 20% between patients and healthy controls or between non-severe ALD and severe AH. ROC analysis shows that seven nucleosides/bases are significantly changed, and all those metabolites have an area under the ROC curve (AUC) larger than 0.7 or smaller than 0.3 (Table 2). Among these significantly changed nucleosides/bases, the abundance (peak area of 2DLC-MS data) of six has a Spearman’s rank correlation coefficient |*ρ*| ≥ 0.4 with MELD score.

**Figure 1:**
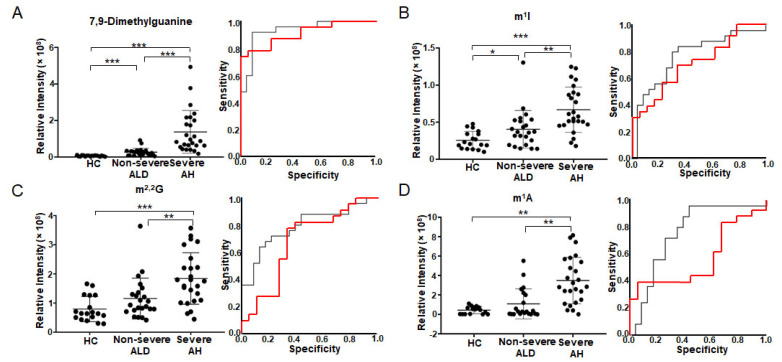
Modified nucleosides/bases with significant abundance change in urine detected by untargeted metabolomics using 2DLC-MS. The scatter plot on the left shows the abundance of 7,9-dimethylguanine (**A**), m^1^I (**B**), m^2,2^G (**C**) and m^1^A (**D**) in urine. The plot on the right shows the ROC analysis results. The red curve shows the ROC analysis results of HC vs. non-severe ALD. The AUC of 7,9-dimethylguanine (**A**) is 0.911, *p* < 0.001 and 95% CI = 0.824–0.998. The AUC of m^1^I (**B**) is 0.710, *p* = 0.022 and 95% CI = 0.553–0.867. AUC of m^2,2^G (**C**) is 0.677, *p* = 0.057 and 95% CI = 0.502–0.852. AUC of m^1^A (**D**) is 0.563, *p* = 0.495 and 95% CI = 0.382–0.744. The black curve shows the ROC analysis result of non-severe ALD vs. severe AH. The AUC of 7,9-dimethylguanine (**A**) is 0.934, *p* < 0.001 and 95% CI = 0.863–1.000. The AUC of m^1^I (**B**) is 0.763, *p* = 0.002 and 95% CI = 0.624–0.903. AUC of m^2,2^G (**C**) is 0.791, *p* = 0.001 and 95% CI = 0.662–0.920. AUC of m^1^A (**D**) is 0.757, *p* = 0.002 and 95% CI = 0.610–0.903. One-way ANOVA was used for the statistical significance test with the Tukey method for post-hoc analysis and the Benjamini and Hochberg method for multiple test correction, * *q* < 0.05; ** *q* < 0.01; *** *q* < 0.001. m^2,2^G, *N*^2^, *N*^2^-dimethylguanosine; m^1^A, 1-methyladenosine; m^1^I, *N*^1^-methylinosine.

Figure 1 depicts the four modified nucleosides/bases with significant abundance change in the urine. These metabolites are listed as the first four metabolites in Table 2. They have the smallest *q*-values in one-way ANOVA (column 3 in Table 2) and the smallest *p*-values and the largest AUC in ROC analysis (columns 4 and 5 in Table 2). All four modified nucleosides/bases have significant abundance changes between HC and patients (Figure 1A–D, left). The abundance of 7,9-dimethylguanine and m^1^I significantly increases from HC to non-severe ALD, and reaches its highest value in severe AH, indicating that these two metabolites can differentiate ALD stages (Figure 1A,B, left). ROC analysis also shows that these two metabolites distinguish not only non-severe ALD from severe AH (AUC between 0.76–0.93) but also non-severe ALD from HC (AUC between 0.71–0.91) (Figure 1A,B, right). Spearman’s rank correlation analysis shows that 7,9-dimethylguanine has the best correlation with MELD score with a correlation coefficient of *ρ* = 0.71, followed by m^1^I with *ρ* = 0.57 (Table 2). These results indicate that these two metabolites are closely related to ALD development.

In addition to 7,9-dimethylguanine and m^1^I, the levels of two other ribonucleosides m^2,2^G (Figure 1C left) and m^1^A (Figure 1D left) are also significantly increased with increasing ALD severity. Their AUCs are between 0.56–0.68 in HC vs. non-severe ALD, and AUC > 0.70 in non-severe ALD vs. severe AH (Figure 1C,D, right), suggesting that those modified ribonucleosides are changed in different stages of ALD. Furthermore, Spearman’s rank correlation coefficient of m^2,2^G with MELD score is 0.48, and m^1^A with MELD score is 0.47 (Table 2).

### 3.2. Some Free Modified Nucleosides/Bases in Patients’ Urine Are Strongly Associated with the Severity of ALD

To verify the 2DLC-MS results of human urine described in Section 3.1, free nucleosides/bases in patients’ urine were further analyzed by targeted metabolomics using a different LC-MS method previously developed by our group [21]. Using that method, nucleosides/bases were enriched by solid phase extraction (SPE) after liquid-liquid extraction. The nucleosides/bases eluted from SPE were then analyzed by RPC-MS. Thirty-nine free nucleosides/bases were detected in urine by SPE RPC-MS. In contrast, 2DLC-MS detected only 25 free nucleosides/bases, demonstrating that the SPE LC-MS platform can minimize the matrix effect and increase the detection limit.

Statistical analysis shows that 14 free nucleosides/bases are significantly changed in the urine of patients with ALD (Table 3), among which 12 are modified nucleosides/bases, 10 have AUC larger than 0.7 or smaller than 0.3, and 6 have Spearman’s rank correlation coefficient |*ρ*| ≥ 0.4 between their abundance and patients’ MELD score. 7,9-Dimethylguanine has the largest Spearman’s rank correlation coefficient of 0.66, followed by 2-methylthio-*N*^6^-threonylcarbamoyladenosine (ms^2^t^6^A) and m^2,2^G (Table 3).

**Figure 2:**
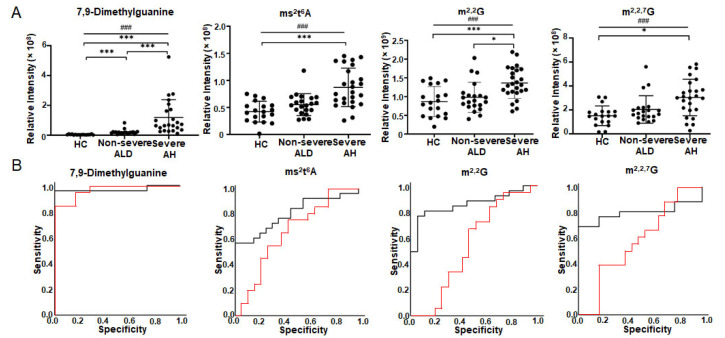
Modified nucleosides/bases with significant abundance change in urine detected by targeted metabolomics using SPE RPC-MS. (**A**) is the abundance changes of modified nucleosides/bases in the urine of patients. (**B**) shows ROC analysis results. The red curve shows the ROC analysis results of HC vs. non-severe ALD. The AUC of 7,9-dimethylguanine is 0.968, *p* < 0.001 and 95% CI = 0.921–1.000. The AUC of ms^2^t^6^A is 0.658, *p* = 0.092 and 95% CI = 0.483–0.833. AUC of m^2,2^G is 0.526, *p* = 0.784 and 95% CI = 0.332–0.721. AUC of m^2,2,7^G is 0.585, *p* = 0.378 and 95% CI = 0.397–0.772. The black curve shows the ROC analysis result of non-severe ALD vs. severe AH. The AUC of 7,9-dimethylguanine is 0.969, *p* < 0.001 and 95% CI = 0.909–1.000. The AUC of ms^2^t^6^A is 0.796, *p* = 0.001 and 95% CI = 0.665–0.927. AUC of m^2,2^G is 0.856, *p* < 0.001 and 95% CI = 0.740–0.971. AUC of m^2,2,7^G is 0.791, *p* = 0.001 and 95% CI = 0.645–0.937. One-way ANOVA was used for the statistical significance test with the Tukey method for post-hoc analysis and the Benjamini and Hochberg method for multiple test correction, * *q* < 0.05; *** *q* < 0.001. The *p*-value scale of the linear-by-linear association test is ^###^ *p* < 0.001. ms^2^t^6^A, 2-methylthio-*N*^6^-threonylcarbamoyladenosine; m^2,2^G, *N*^2^, *N*^2^-dimethylguanosine; m^2,2,7^G*, N*^2^,*N*^2^,7-trimethylguanosine.

Figure 2 depicts the four metabolites that have *q*-value < 0.05 in one-way ANOVA and the best AUC in ROC analysis (Table 3). The abundance of 7,9-dimethylguanine increases from HC to non-severe ALD and reaches its highest value in severe AH. The abundance changes of this metabolite can differentiate HC from non-severe ALD, and non-severe ALD from severe AH (Figure 2A). Furthermore, the abundance changes of m^2,2^G can differentiate severe AH from non-severe ALD and HC (Figure 2A). These results are consistent with 2DLC-MS (Figure 1 and Table 2). However, two metabolites ms^2^t^6^A and *N*^2^,*N*^2^,7-trimethylguanosine (m^2,2,7^G) were detected by SPE RPC-MS, but not by 2DLC-MS. The increase of ms^2^t^6^A and m^2,2,7^G only has statistical significance between severe AH and HC (Figure 2A). Linear-by-linear association test shows a statistically significant trend for all four modified nucleosides/bases (Figure 2A), suggesting that the levels of these nucleosides/bases have an ascending trend with the increase in disease severity.

Figure 2B shows the ROC analysis results of the four modified nucleosides/bases depicted in Figure 2A. 7,9-Diemthylguanine can distinguish severe AH from non-severe ALD (AUC = 0.97 with 95% CI from 0.91 to 1.00) and non-severe ALD from HC (AUC = 0.97 with 95% CI from 0.92 to 1.00). m^2,2^G, ms^2^t^6^A, and m^2,2,7^G can differentiate non-severe ALD from severe AH (AUC between 0.79–0.86), but they cannot distinguish non-severe ALD from HC (AUC between 0.53–0.66).

The similar results of 7,9-dimethylguanine analyzed using three different analysis methods, i.e., one-way ANOVA, linear-by-linear association test, and ROC analysis, suggest that 7,9-dimethylguanine in urine is strongly correlated to the severity of ALD. Free-modified ribonucleosides/bases are degradation products of RNA. 7,9-dimethylguanine is a rare nucleobase in tRNA and the cap of mRNA [26,27]. The abundance change of 7,9-dimethylguanine in the urine of patients with ALD indicates the alteration of RNA metabolism, which may be a new mechanism of ALD development/progression.

### 3.3. Modified Nucleosides/Bases Are Changed in Serum of Patients with ALD

We also analyzed the free nucleosides/bases in human serum by SPE RPC-MS. Forty free nucleosides/bases were detected. Unfortunately, 7,9-dimethylguanine was not detectable owing to the low intensity of this metabolite in serum. One-way ANOVA shows 13 detected ribonucleosides are significantly changed, and 11 are modified (Table 4). ROC analysis shows 9 modified ribonucleosides (Table 4) have an AUC larger than 0.7 or smaller than 0.3, and 7 have statistical significance with *p* < 0.05.

**Figure 3:**
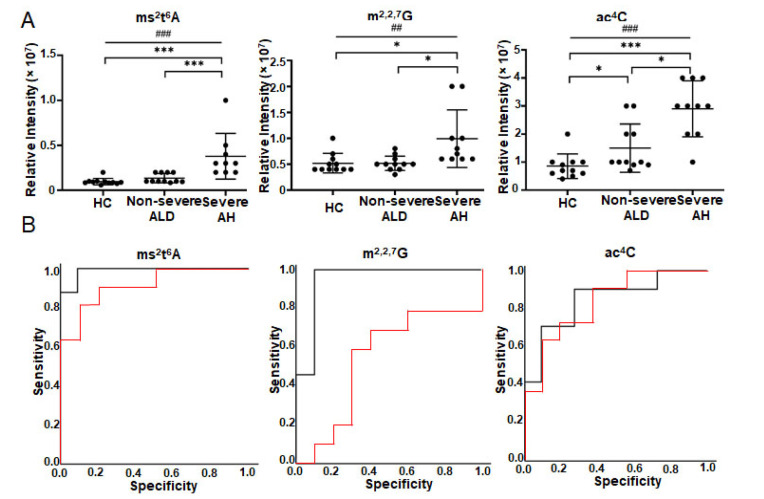
Modified ribonucleoside changes in the serum of the patients with ALD detected by targeted metabolomics using SPE RPC-MS. Nucleosides were extracted from the serum of healthy volunteers (*n* = 11) and patients (*n* = 23) and detected by SPE LC-MS under positive mode. (**A**) is the abundance changes of modified ribonucleosides in the serum of patients. (**B**) shows ROC analysis results. The red curve shows the ROC analysis results of HC vs. non-severe ALD. The AUC of ms^2^t^6^A is 0.918, *p* = 0.001 and 95% CI = 0.801–1.000. AUC of m^2,2,7^G is 0.550, *p* = 0.705 and 95% CI = 0.280–0.820. AUC of ac^4^C is 0.843 *p* = 0.006 and 95% CI = 0.680–1.000. The black curve shows the ROC analysis result of non-severe ALD vs. severe AH. The AUC of ms^2^t^6^A is 0.989, *p* < 0.001 and 95% CI = 0.953–1.000. AUC of m^2,2,7^G is 0.944, *p* = 0.001 and 95% CI = 0.932–1.000. AUC of ac^4^C is 0.845 *p* = 0.007 and 95% CI = 0.672–1.000. One-way ANOVA was used for the statistical significance test with the Tukey method for post-hoc analysis and the Benjamini and Hochberg method for multiple test correction, * *q* < 0.05; *** *q* < 0.001. The *p*-value scale of the linear-by-linear association test is ^##^ *p* < 0.01; ^###^ *p* < 0.001. ms^2^t^6^A, 2-methylthio-*N*^6^-threonylcarbamoyladenosine; ac^4^C, *N*^4^-acetylcytidine; m^2,2,7^G*, N*^2^,*N*^2^,7-trimethylguanosine.

Figure 3 depicts three metabolites that have *q*-value < 0.05 in one-way ANOVA and the best AUC in ROC analysis (lines 1–3 in Table 4). The abundance increase of *N*^4^-acetylcytidine (ac^4^C) can differentiate all three groups, while the abundance changes of m^2,2,7^G and ms^2^t^6^A can differentiate severe AH from non-severe ALD and HC (Figure 3A). Linear-by-linear association test shows a statistically significant ascending trend for all three modified ribonucleosides with ALD severity (Figure 3A). Spearman’s rank correlation analysis indicates that ms^2^t^6^A has the highest correlation coefficient with MELD score (*ρ* = 0.74), followed by ac^4^C and m^2,2,7^G (column 13 in Table 4). ROC analysis shows that ms^2^t^6^A is the best ribonucleoside in serum to distinguish severe AH and non-severe ALD with an AUC of 0.99 (95% CI = 0.95 to 1.00), followed by m^2,2,7^G (AUC = 0.94 with 95% CI = 0.83 to 1.00) and ac^4^C (AUC = 0.85 with 95% CI = 0.67 to 1.00) (Figure 3B). ms^2^t^6^A (AUC = 0.92 with 95% CI from 0.80 to 1.00) and ac^4^C (AUC = 0.84 with 95% CI from 0.68 to 1.00) can also differentiate non-severe ALD from HC, but m^2,2,7^G can not (AUC = 0.55 with 95% CI = 0.28–0.82) (Figure 3B).

## 4. Discussion

This study aimed to investigate the abundance changes of free-modified nucleosides/bases in the urine and serum of patients with ALD and determine whether these metabolites are associated with the severity of ALD. Our results show that modified nucleosides/bases are changed in the urine and serum of patients with ALD. Twelve modified nucleosides/bases in urine have significant abundance alteration between groups, while eleven have substantial abundance changes in serum. m^2,2^G, ms^2^t^6^A, and m^2,2,7^G have significant abundance changes in both urine and serum, and their abundance has a moderate correlation with MELD score (Table 3 and Table 4). However, 7,9-dimethylguanine has the largest abundance alteration in urine, and ms^2^t^6^A has the largest abundance alteration in serum. The abundance of 7,9-dimethylguanine in urine (Figure 1 and Figure 2) and ms^2^t^6^A in serum (Figure 3) are significantly increased with disease severity, and the abundance of each strongly correlates with patients’ MELD score, indicating that these two metabolites are related to ALD disease development. Besides the MELD score, 7,9-dimethylguanine in urine also has a very good correlation with bilirubin (*ρ* = 0.64) and a moderate correlation with alkaline phosphatase (*ρ* = 0.45) and albumin (*ρ* = −0.44) (data not shown), suggesting a strong relationship of 7,9-dimethylguanine with ALD disease development. Three statistical analyses, i.e., one-way ANOVA, linear-by-linear association test, and ROC analysis, show using the abundance of 7,9-dimethylguanine and ms^2^t^6^A can differentiate healthy people from patients with non-severe ALD and patients with non-severe ALD from those with severe AH.

7,9-Dimethylguanine is a rare nucleobase in the cap of message RNA (mRNA) and transfer RNA (tRNA) [26,27] 7,9-Dimethylguanine is converted from *N*^7^-methylguanine [28], which has various functions in an organism. *N*^7^-Methylguanine in the 5’ cap of mature mRNAs mediates cap-related biological functions and functions as an identifier of self RNA in the innate immune system, then ensures cap-dependent protein synthesis [29]. The conversion of *N*^7^-methylguanine to 7,9-dimethylguanine changes cap-related mRNA stability, splicing, and protein synthesis, resulting in biased gene expression. tRNA acts as the adapter molecule of amino acids and is a key component in protein synthesis. Chemical modifications on tRNA contribute to codon-anticodon interactions. Conversion of *N*^7^-methylguanine to 7,9-dimethylguanine on tRNA may change the structure and stability of tRNA, resulting in the inaccuracy of translation. 7,9-Dimentylguanine also exists in DNA which causes homo GG pairing [27,30] and then changes the RNA sequence and gene expression. However, no further studies show the mechanism of 7,9-dimethylguanine in biological processes, including the development of ALD.

ms^2^t^6^A exists in tRNA and usually happens in the position of 37 [31]. Modification of tRNA in position 37 is critical for structuring the anticodon loop and enhancing the ability of tRNA to bind to poly-A programmed ribosomes [32]. The methylthiolation of A^37^ in tRNA prevents the misreading and frame-shifting of cognate codons during translation and ensures decoding fidelity. Changes in tRNA A^37^ modification may cause mistranslation of the condon, even disease development. ms^2^t^6^A is a modified nucleoside in tRNA anticodon loops. Studies on ms^2^t^6^A modification in position 37 of tRNA^lys^ show an important role of this modification in preventing the misreading of its cognate codons, especially when the rate of translation is relatively high [33]. Deficiency of ms^2^t^6^A^37^ modification in tRNA^lys^ (UUU) causes incorrectly translating the insulin mRNA AAG codon for lysine at the site of protease cleavage between the A-chain and the C-peptide [34] and results in the development of type 2 diabetes [33]. Our previous study also shows that ms^2^t^6^A was significantly decreased in mouse liver after polychlorinated biphenyls exposure [20], suggesting that ms^2^t^6^A plays a role in liver disease development.

In this study, we found that 7,9-dimethylguanine has the most distinguished increase in the urine and ms^2^t^6^A in the serum of patients with ALD, indicating that these two modifications may contribute to ALD development and progression. The increase of these modified nucleosides/bases may change the translation rate or accuracy of mRNA when the modification happens in the 5’ cap of mRNA or tRNA, thus causing different protein expressions in the livers of patients with ALD. It has been reported that some modified nucleosides/bases discovered in this study have altered abundance in patients with different liver diseases [20,35]. Still, none of these studies investigated the function of these modified nucleosides in disease development. Therefore, extensive study is needed to determine the role of these RNA modifications in ALD development.

This study has some limitations. We included 46 patients, and a larger validation cohort should be conducted in the future. We did not evaluate other types of liver disease, such as viral liver disease. We based the severity of liver injury on the MELD score and did not have liver biopsies. Moreover, we used urine and serum samples with no human liver tissue and can not detect the level of these modifications on the RNA level. Therefore, the source of these free modified nucleosides/bases is unclear. Importantly, liver biopsies are only infrequently performed for the diagnosis of ALD in the United States. Lastly, there are no longitudinal data or functional correlates of RNA modification, and obtaining these data are the goal for future studies.

## 5. Conclusions

We analyzed the free nucleosides/bases in the urine and serum of patients with ALD. The abundance of modified nucleosides/bases, notably 7,9-dimethylguanine and ms^2^t^6^A are respectively increased in urine and serum. The elevation of these two modified nucleosides/bases is closely related to ALD disease severity, and the abundance difference of these metabolites is sufficiently large to distinguish different stages of ALD.

## Figures and Tables

**Table 1:** Demographic, and liver indicators of study participants by group.

Variables	HC (*n* = 19)	Alcohol-Associated Liver Disease (ALD)				*p*-Value ^a^
		Non-Severe ALD (*n* = 21)		Severe AH (*n* = 25)	Total Patients (*n* = 46)	
		AUD (*n* = 8)	Moderate AH (n = 13)			
Age (years)	36 (24–60)	51 (39–67)	50 (34–65)	47 (27–66)	49 (27–67)	-
Male (Female)	13 (6)	4 (4)	7 (6)	20 (5)	31 (15)	-
BMI	N/A	31.1 (27.8–33.4)	25.9 (20.6–41.1)	29.7 (22.4–50.5)	29.7 (20.6–50.5)	-
T. Bilirubin (mg/dL)	0.7 (0.4–1.3)	1.5 (0.8–2.6)	4.2 (1.2–18.2)	12.9 (3.7–34.2)	6.7 (0.8–34.2)	<0.001
INR	N/A	1.2 (1.1–1.7)	1.5 (1.2–2.8)	2.0 (1.0–3.2)	1.7 (1.0–3.2)	-
AST (U/L)	27 (19–66)	59 (21–120)	119 (53–347)	88 (16–190)	90 (16–347)	<0.001
ALT (U/L)	25 (16–109)	36.8 (14.0–60.0)	48 (18–194)	35 (16–66)	39 (14–194)	0.015
Alkaline phosphatase (IU/L)	52 (37–62)	124 (89–232)	173 (80–518)	144 (71–336)	148 (71–518)	<0.001
Albumin (g/dL)	4.2 (3.8–4.3)	3.9 (2.6–4.9)	2.8 (1.9–4.5)	2.4 (1.4–4.3)	2.7 (1.4–4.9)	<0.001
Creatinine (mg/dL)	0.88 (0.69–1.07)	0.69 (0.36–1.40)	0.68 (0.32–1.30)	0.89 (0.39–5.68)	0.79 (0.32–5.68)	0.082
MELD score	N/A	9.2 (6.0–11.0)	16 (12–19)	24 (20–39)	18 (6–39)	-

Values are presented as mean with ranges. HC, healthy controls; ALD, alcohol-associated liver disease; AUD, alcohol use disorder; AH, alcohol-associated hepatitis; ALT, alanine aminotransferase; AST, aspartate aminotransferase; INR, international normalized ratio; BMI, body mass index; MELD, model for end-stage liver disease; N/A, not available; ^a^: Mann–Whitney U test between healthy controls and alcoholic patients.

**Table 2:** Nucleosides/bases with significant abundance changes between groups in human urine.

Name	*t_R_* (min)	*q*-Value ^a^	ROC Analysis			Non-Severe AH vs. HC		Severe AH vs. HC		Severe AH vs. Non-Severe ALD		ρ-Value ^e^
			*p*-Value ^b^	AUC ^c^	95% CI ^c^	Fold-Change ^d^	*q*-Value ^a^	Fold-Change ^d^	*q*-Value ^a^	Fold-Change ^d^	*q*-Value ^a^	
7,9-dimethylguanine	6.35	<0.001	<0.001	0.934	0.863–1.000	3.20	<0.001	18.63	<0.001	5.82	<0.001	0.71
m^2,2^G	5.52	<0.001	0.001	0.791	0.662–0.920	1.31	0.071	2.32	<0.001	1.77	0.008	0.48
m^1^A	6.68	0.001	0.002	0.757	0.610–0.903	2.47	0.774	7.99	0.001	3.24	0.004	0.47
m^1^I	5.42	<0.001	0.002	0.763	0.624–0.903	1.42	0.043	2.62	<0.001	1.84	0.002	0.57
m^3^C/m^4^C/m^5^C	6.86	0.007	0.003	0.235	0.100–0.370	1.08	0.547	0.24	0.094	0.22	0.003	−0.48
C	9.98	<0.001	0.003	0.752	0.609–0.896	0.97	0.208	2.63	<0.001	2.72	0.048	-
DHU	6.64	0.001	0.003	0.748	0.610–0.886	1.11	0.767	3.15	0.001	2.85	0.005	0.45
1-Methylguanine	9.62	0.033	-	-	-	1.12	0.189	2.27	0.033	2.04	0.192	-
A	5.72	0.039	-	-	-	0.93	0.664	0.66	0.039	0.71	0.201	-
Uracil	6.58	0.034	-	-	-	2.08	0.086	3.35	0.034	1.61	0.921	-

^a^: One-way ANOVA with the Tukey method for post-test and the Benjamini and Hochberg method for multiple test correction. ^b^: ROC analysis of non-severe ALD with severe AH. ^c^: AUC (area under the ROC curve) and asymptotic 95% confidence interval of ROC. AH, alcohol-associated hepatitis; HC, healthy controls; ALD, alcohol-associated liver disease. ^d^: Fold-change is the ratio of the mean peak area of a metabolite in the first group divided by that in the second group. ^e^: Spearman’s rank correlation coefficient. “-”: Value does not meet the threshold. m^2,2^G, *N*^2^, *N*^2^-dimethylguanosine; m^1^A, 1-methyladenosine; m^1^I, *N*^1^-methylinosine; m^3^C, 3-methylcytidine; m^4^C, *N*^4^-methylcytidine; m^5^C, 5-methylcytidine; C, cytidine; DHU, dihydrouridine; A, adenosine.

**Table 3:** Significantly changed nucleosides in human urine samples.

Name	*t_R_* (min)	*q*-Value ^a^	ROC Analysis			Non-Severe ALD vs. HC		Severe ALD vs. HC		Severe AH vs. Non-Severe ALD		ρ-Value ^e^
			*p*-Value ^b^	AUC ^c^	95% CI ^c^	Fold-Change ^d^	*q*-Value ^a^	Fold-Change ^d^	*q*-Value ^a^	Fold-Change ^d^	*q*-Value ^a^	
7,9-Dimethylguanine	4.95	<0.001	<0.001	0.969	0.909–1.000	5.35	<0.001	19.20	<0.001	3.59	<0.001	0.66
ms^2^t^6^A	18.98	<0.001	0.001	0.796	0.665–0.927	1.28	0.314	1.86	<0.001	1.45	0.119	0.50
m^2,2^G	10.85	<0.001	<0.001	0.856	0.740–0.971	1.02	0.974	1.56	<0.001	1.53	0.013	0.50
m^2,2,7^G	9.60	0.013	0.001	0.791	0.645–0.937	1.09	0.691	2.02	0.013	1.86	0.161	-
5′-Deoxyuridine	4.26	0.031	0.006	0.738	0.591–0.885	1.09	0.931	1.65	0.031	1.50	0.130	0.47
DHU	4.27	0.009	0.006	0.737	0.589–0.885	1.10	0.691	1.67	0.009	1.51	0.130	0.44
MTA	12.84	0.009	0.011	0.737	0.582–0.891	1.43	0.818	2.54	0.009	1.77	0.111	-
mcm^5^s^2^U	12.92	0.027	0.002	0.779	0.636–0.922	0.78	0.999	1.43	0.092	1.84	0.027	-
m^1^I	8.91	<0.001	0.017	0.713	0.555–0.871	1.16	0.136	1.47	<0.001	1.27	0.284	0.48
m^6^A	9.15	0.073	0.005	0.233	0.062–0.404	1.59	1.000	0.72	0.130	0.45	0.073	-
m^3^U	9.01	0.136	0.016	0.719	0.559–0.880	0.89	0.776	1.14	0.663	1.28	0.136	-
I	7.12	0.018	-	-	-	1.72	0.018	1.93	0.119	1.12	0.875	-
Ψ	4.36	0.013	-	-	-	1.04	0.139	1.52	0.013	1.46	0.728	-
G	7.10	0.009	-	-	-	1.62	0.300	2.08	0.009	1.28	0.330	-

^a^: One-way ANOVA with the Tukey method for post-test and the Benjamini and Hochberg method for multiple test correction. ^b^: ROC analysis of non-severe ALD with severe AH. ^c^: AUC (area under the ROC curve) and asymptotic 95% confidence interval of ROC. AH, alcohol-associated hepatitis; HC, healthy controls; ALD, alcohol-associated liver disease. ^d^: Fold-change is the ratio of the mean peak area of a metabolite in the first group divided by that in the second group. ^e^: Spearman’s rank correlation coefficient. “-”: Value is larger than the threshold. ms^2^t^6^A, 2-methylthio-*N*^6^-threonylcarbamoyladenosine; m^2,2^G, *N*^2^, *N*^2^-dimethylguanosine; m^2,2,7^G, *N*^2^,*N*^2^,7-trimethylguanosine; DHU, dihydrouridine; MTA, 5’-s-methyl-5’-thioadenosine; mcm^5^s^2^U, 5-methoxycarbonylmethyl-2-thiouridine; m^1^I, *N*^1^-methylinosine; m^6^A, *N*^6^-methyladenosine; m^3^U, 3-methyluridine; I, inosine; Ψ, pseudouridine; G, guanosine.

**Table 4:** Significantly changed ribonucleosides in human serum samples.

Name	*t_R_* (min)	*q*-Value ^a^	ROC Analysis		Non-Severe ALD vs. HC		Severe ALD vs. HC		Severe AH vs. Non-Severe ALD			ρ-Value ^e^
			*p*-Value ^b^	AUC ^c^	95% CI ^c^	Fold-Change ^d^	*q*-Value ^a^	Fold-Change ^d^	*q*-Value ^a^	Fold-Change ^d^	*q*-Value ^a^	
ms^2^t^6^A	19.01	<0.001	<0.001	0.989	0.953–1.000	1.62	0.173	2.97	<0.001	1.83	<0.001	0.74
ac^4^C	9.63	<0.001	0.007	0.845	0.672–1.000	1.86	0.036	3.99	<0.001	2.15	0.028	0.59
m^2,2,7^G	9.55	0.022	0.001	0.944	0.832–1.000	1.23	1.000	1.75	0.022	1.43	0.022	0.50
m^2,2^G	10.84	0.033	0.102	0.722	0.469–0.975	1.21	0.265	1.54	0.033	1.28	0.494	-
m^1^I	8.89	<0.001	0.014	0.828	0.647–1.000	1.36	0.265	1.93	<0.001	1.42	0.040	0.47
m^3^U	8.98	0.018	0.014	0.828	0.644–1.000	1.03	1.000	1.70	0.018	1.66	0.022	0.44
m^5^U	7.88	0.179	0.029	0.218	0.007–0.429	0.97	1.000	0.79	0.362	0.82	0.179	-
ncm^5^U	5.56	0.010	-	0.727	0.502–0.953	1.08	0.531	1.51	0.010	1.39	0.129	0.44
m^4^C	5.18	0.173	0.017	0.170	0.000–0.364	1.65	0.316	0.77	1.000	0.47	0.173	−0.40
C	4.25	<0.001	0.007	0.860	0.689–1.000	1.95	0.087	4.06	<0.001	2.08	0.086	0.67
I	7.09	<0.001	-	-	-	0.14	<0.001	0.13	<0.001	0.91	1.000	-
m^5^Um	10.96	0.010	-	-	-	1.53	0.071	1.72	0.010	1.13	0.577	-
G	7.08	<0.001	-	-	-	0.09	<0.001	0.10	<0.001	1.10	1.000	-

^a^: One-way ANOVA with the Tukey method for post-test and the Benjamini and Hochberg method for multiple test correction. ^b^: ROC analysis of non-severe ALD with severe AH. ^c^: AUC (area under the ROC curve) and asymptotic 95% confidence interval of ROC. AH, alcohol-associated hepatitis; HC, healthy controls; ALD, alcohol-associated liver disease. ^d^: Fold-change is the ratio of the mean peak area of a metabolite in the first group divided by that in the second group. ^e^: Spearman’s rank correlation coefficient. “-”: Value does not meet the threshold. ms^2^t^6^A, 2-methylthio-*N*^6^-threonylcarbamoyladenosine; ac^4^C, *N*^4^-acetylcytidine; m^2,2^G, *N*^2^, *N*^2^-dimethylguanosine; m^2,2,7^G*, N*^2^,*N*^2^,7-trimethylguanosine; m^1^I, *N*^1^-methylinosine; m^3^U, 3-methyluridine; m^5^U, 5-methyluridine; ncm^5^U, 5-carbamoylmethyluridine; m^4^C, *N*^4^-methylcytidine; C, cytidine; I, inosine; m^5^Um, 5,2’-o-dimethyluridine; G, guanosine.

## Data Availability

The data presented in this study are available in the main article.
